# Adverse outcomes and an immunosuppressed endotype in septic patients with reduced IFN-**γ** ELISpot

**DOI:** 10.1172/jci.insight.175785

**Published:** 2024-01-23

**Authors:** Evan L. Barrios, Monty B. Mazer, Patrick W. McGonagill, Christian B. Bergmann, Michael D. Goodman, Robert W. Gould, Mahil Rao, Valerie E. Polcz, Ruth J. Davis, Drew E. Del Toro, Marvin L.S. Dirain, Alexandra Dram, Lucas O. Hale, Mohammad Heidarian, Caleb Y. Kim, Tamara A. Kucaba, Jennifer P. Lanz, Ashley E. McCray, Sandra Meszaros, Sydney Miles, Candace R. Nelson, Ivanna L. Rocha, Elvia E. Silva, Ricardo F. Ungaro, Andrew H. Walton, Julie Xu, Leilani Zeumer-Spataro, Anne M. Drewry, Muxuan Liang, Letitia E. Bible, Tyler J. Loftus, Isaiah R. Turnbull, Philip A. Efron, Kenneth E. Remy, Scott C. Brakenridge, Vladimir P. Badovinac, Thomas S. Griffith, Lyle L. Moldawer, Richard S. Hotchkiss, Charles C. Caldwell

**Affiliations:** 1 Sepsis and Critical Illness Research Center, Department of Surgery, University of Florida College of Medicine, Gainesville, Florida, USA.; 2 Department of Pediatrics, Case Western Reserve University School of Medicine, Cleveland, Ohio, USA.; 3 Department of Surgery, University of Iowa Carver College of Medicine, Iowa City, Iowa, USA.; 4 Department of Surgery, University of Cincinnati College of Medicine, Cincinnati, Ohio, USA.; 5 University Hospital Ulm, Clinic for Trauma Surgery, Hand, Plastic, and Reconstructive Surgery Albert-Einstein-Allee 23, Ulm, Germany.; 6 Department of Anesthesiology, University of Minnesota Medical School, Minneapolis, Minnesota, USA.; 7 Department of Pediatrics, University of Iowa Carver College of Medicine, Iowa City, Iowa, USA.; 8 Department of Anesthesiology, Washington University School of Medicine, St. Louis, Missouri, USA.; 9 Interdisciplinary Program in Immunology, University of Iowa Carver College of Medicine, Iowa City, Iowa, USA.; 10 Department of Urology, University of Minnesota Medical School, Minneapolis, Minnesota, USA.; 11 Department of Pathology, University of Iowa Carver College of Medicine, Iowa City, Iowa, USA.; 12 Department of Biostatistics, University of Florida College of Public Health and Health Professions and the University of Florida College of Medicine, Gainesville, Florida, USA.; 13 Department of Surgery, Harborview Medical Center, University of Washington School of Medicine, Seattle, Washington, USA.; 14 Experimental Pathology PhD Program, University of Iowa Carver College of Medicine, Iowa City, Iowa, USA.; 15 Center for Immunology, University of Minnesota Medical School, Minneapolis, Minnesota, USA.; 16 Minneapolis VA Healthcare System, Minneapolis, Minnesota, USA.

**Keywords:** Immunology, Adaptive immunity, Cellular immune response, T cells

## Abstract

**BACKGROUND:**

Sepsis remains a major clinical challenge for which successful treatment requires greater precision in identifying patients at increased risk of adverse outcomes requiring different therapeutic approaches. Predicting clinical outcomes and immunological endotyping of septic patients generally relies on using blood protein or mRNA biomarkers, or static cell phenotyping. Here, we sought to determine whether functional immune responsiveness would yield improved precision.

**METHODS:**

An ex vivo whole-blood enzyme-linked immunosorbent spot (ELISpot) assay for cellular production of interferon γ (IFN-γ) was evaluated in 107 septic and 68 nonseptic patients from 5 academic health centers using blood samples collected on days 1, 4, and 7 following ICU admission.

**RESULTS:**

Compared with 46 healthy participants, unstimulated and stimulated whole-blood IFN-γ expression was either increased or unchanged, respectively, in septic and nonseptic ICU patients. However, in septic patients who did not survive 180 days, stimulated whole-blood IFN-γ expression was significantly reduced on ICU days 1, 4, and 7 (all *P* < 0.05), due to both significant reductions in total number of IFN-γ–producing cells and amount of IFN-γ produced per cell (all *P* < 0.05). Importantly, IFN-γ total expression on days 1 and 4 after admission could discriminate 180-day mortality better than absolute lymphocyte count (ALC), IL-6, and procalcitonin. Septic patients with low IFN-γ expression were older and had lower ALCs and higher soluble PD-L1 and IL-10 concentrations, consistent with an immunosuppressed endotype.

**CONCLUSIONS:**

A whole-blood IFN-γ ELISpot assay can both identify septic patients at increased risk of late mortality and identify immunosuppressed septic patients.

**TRIAL REGISTRY:**

N/A.

**FUNDING:**

This prospective, observational, multicenter clinical study was directly supported by National Institute of General Medical Sciences grant R01 GM-139046, including a supplement (R01 GM-139046-03S1) from 2022 to 2024.

## Abstract 2

<p>IFN&gamma; ELISpot predicts long-term survival in sepsis patients when measured within 4-7 days of ICU admission.</p>

## Graphical abstract

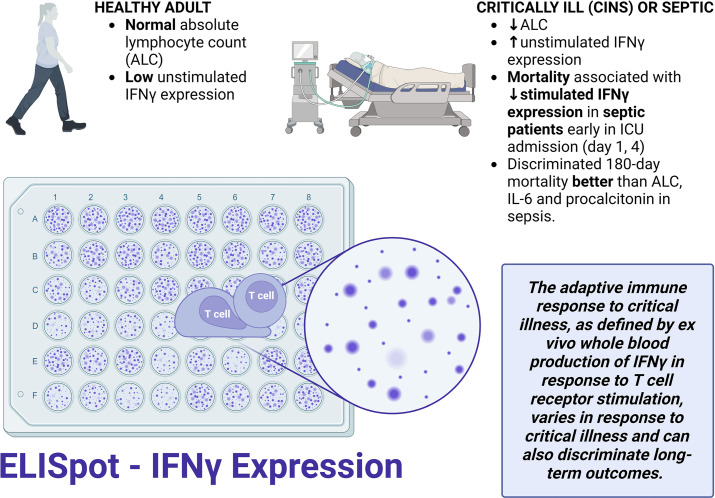

## Introduction

Sepsis remains one of the most common causes of critical illness and too often leads to death and morbidity (1–3). Importantly, sepsis is a pathophysiologic host response to microbial infection associated with organ injury and dysfunction (4). However, the nature and magnitude of the host response to sepsis is highly variable, depending on the individual’s age, comorbidities, and source and severity of microbial infection. Although sepsis is frequently associated with an early exaggerated inflammatory response (5), persistent inflammation (6, 7), coagulopathy (8), prolonged immunosuppression (3, 6, 9–12), and lean tissue wasting (13, 14), the contribution of these individual responses to the overall outcome of the patient is still unresolved (15, 16). Precision medicine has been proposed as a tool to identify which immunologic endotype drives organ injury and is an appropriate target for therapeutic intervention (17). Biomarkers, based on static blood cell phenotypes, protein, and transcriptomic metrics, have been commonly used to endotype critically ill patients with and without sepsis (15, 17–20).

The enzyme-linked immunosorbent spot (ELISpot) assay is a widely used immunological technique that enables the detection and quantification of individual cells responding to external receptor-specific and nonspecific stimulants and secreting specific proteins, particularly cytokines (21). This method is important for studying the immune response at the single-cell level, offering valuable insights into immune cell function and immune-related diseases. Its ability to analyze immune responses at the cellular level makes it particularly suitable for monitoring immune system functionality in sepsis. In the current report, we examined the extent to which whole-blood ELISpot production of interferon γ (IFN-γ) can identify immunosuppressed, critically ill patients at increased risk of death.

## Results

### Patient characteristics.

Demographic characteristics of the 175 enrolled patients (99 men [57%] and 76 women [43%]) and 46 healthy control participants (16 men [36%], 30 women [64%]) are summarized in Table 1. The overall cohort included 107 and 68 patients with a suspected diagnosis of sepsis admitted to the surgical/trauma ICU (SEPSIS) and critically ill, nonseptic (CINS) cohorts, respectively. Consistent with NIH reporting requirements, 80%, 76%, and 78% of the SEPSIS, CINS, and healthy participants, respectively, defined themselves as White; 14%, 19%, and 7% as African-American; 0%, 3%, and 13% as multiracial; and less than 1% as either Native American, Pacific Islander, or Asian, with the exception of healthy participants, among which 9% defined themselves as Asian. In addition, 97%, 100%, and 87% of the SEPSIS, CINS, and healthy participants, respectively, defined themselves as non-Hispanic.

Patient characteristics were similar across all 3 cohorts, with the exception that the healthy control participants were more predominantly female and younger, and SEPSIS patients had a higher Charlson comorbidity index than CINS participants (Table 1). Within the CINS cohort (*n* = 68), the reasons for ICU admission are identified in Supplemental Table 1; supplemental material available online with this article; https://doi.org/10.1172/jci.insight.175785DS1

Table 2 shows clinical outcomes for the SEPSIS and CINS patients. Hospital length of stay (*P* < 0.02), incidence of secondary infections (*P* < 0.001), development of chronic critical illness (CCI) (*P* < 0.001), and in-hospital mortality (*P* < 0.01) were all significantly higher in SEPSIS than in CINS patients. Disposition at discharge also significantly differed between SEPSIS and CINS patients, as did 30- and 180-day mortality (both *P* < 0.01).

### IFN-γ production by unstimulated and anti-CD3/anti-CD28 mAb–stimulated whole blood.

**Figure 1:**
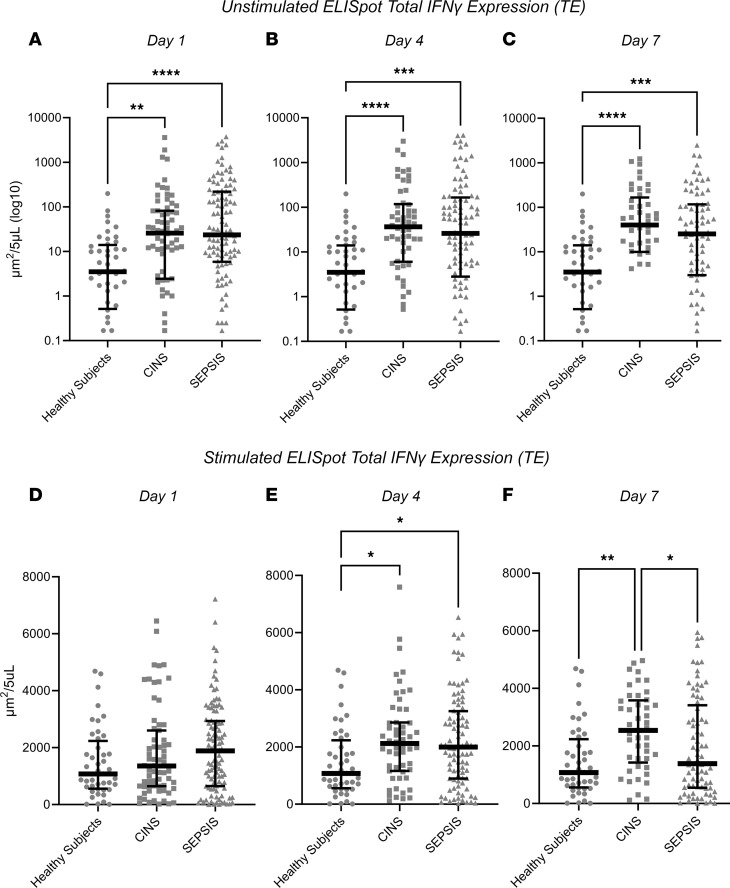
Unstimulated and stimulated IFN-γ expression as determined by ELISpot in SEPSIS and CINS patients and healthy control participants on days 1, 4, and 7 following ICU admission. Values represent medians and individual subject responses. (**A**–**C**) IFN-γ expression in unstimulated whole blood. (**D**–**F**) IFN-γ expression in anti-CD3/anti-CD28–stimulated whole blood. Note that the scales for unstimulated expression are logarithmic, whereas they are linear for stimulated expression to appropriately reflect the magnitude and heterogeneity of the individual response. *P < 0.05, **P < 0.01, ***P < 0.001, ****P < 0.0001, as determined by Kruskal-Wallis ANOVA and post hoc analyses using Dunn’s test. Values are 2 sided and represent raw *P* values. SFU, spot-forming units; SS, spot size; TE, total IFN-γ expression.

**Figure 2:**
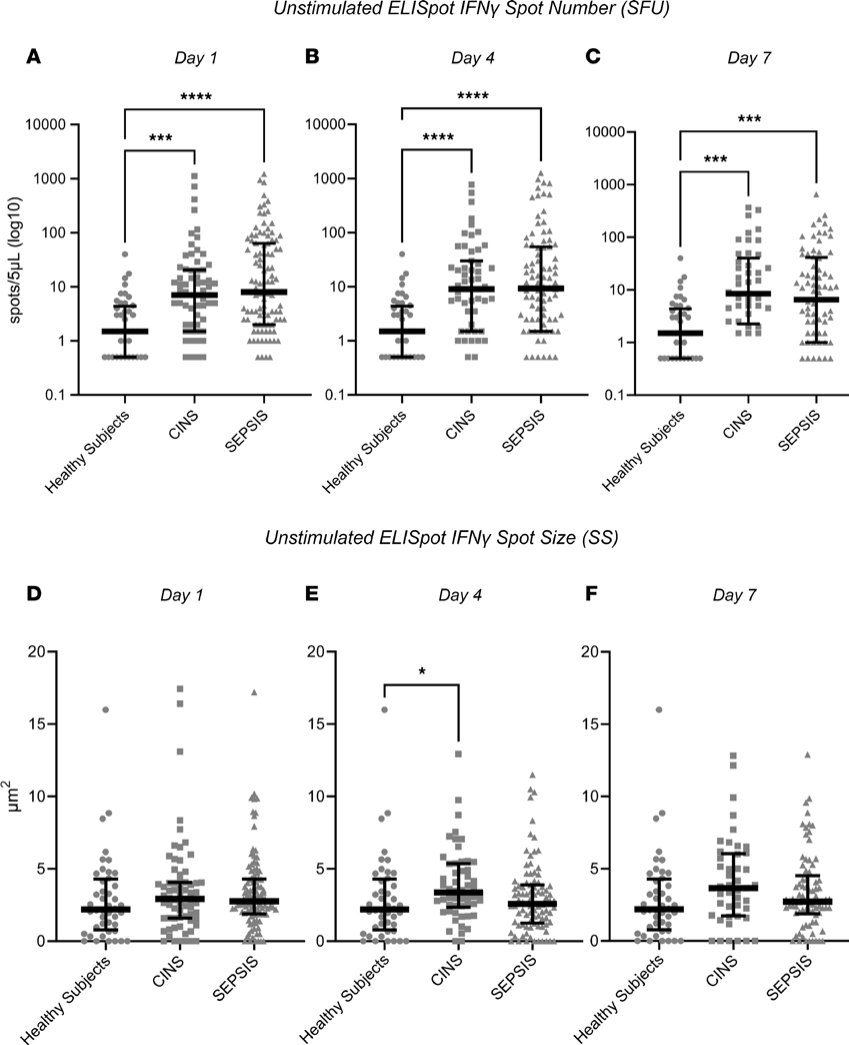
ELISpot SFU and SS from unstimulated whole blood in the 3 cohorts (healthy participants, SEPSIS, and CINS). (**A**–**C**) IFN-γ SFU. (**D**–**F**) IFN-γ SS. In unstimulated whole blood, SEPSIS and CINS cohorts demonstrated a consistent increase in the number of cells (SFU) producing IFN-γ, when compared with healthy control participants. *P < 0.05, ***P < 0.001, ****P < 0.0001, as determined by Kruskal-Wallis ANOVA and post hoc analyses using Dunn’s test. Values are 2 sided and represent raw *P* values. SFU, spot-forming units; SS, spot size; TE, total IFN-γ expression.

Comparison of the first sample collected (days 1–3 after ICU admission) among the 3 cohorts revealed considerable heterogeneity in the individual response, irrespective of the cohort. Surprisingly, as a group, spontaneous IFN-γ production in unstimulated whole blood was significantly increased from SEPSIS and CINS patients compared with healthy participants at all sampling intervals (days 1, 4, and 7), despite a significant reduction in lymphocyte numbers (Figure 1 and Supplemental Figure 1). This was reflected generally by an increased number of IFN-γ–producing cells (spot-forming units [SFU]) (all *P* < 0.05), although the amount of IFN-γ produced by each cell (spot size [SS]) was increased on day 4 (Figure 2). When the total number of IFN-γ–producing cells was adjusted for the absolute lymphocyte count (ALC), the percentage of lymphocytes expressing IFN-γ was further increased significantly in both SEPSIS and CINS (*P* < 0.001; Supplemental Figure 2). There was no difference in unstimulated IFN-γ expression between the SEPSIS and CINS cohorts.

Ex vivo stimulation of the whole blood from the 3 cohorts with agonistic anti-CD3/anti-CD28 mAb resulted in expected increases in the total expression (TE) of IFN-γ produced when compared with unstimulated samples. There were also increase in the total amount of IFN-γ produced per unit volume of blood on days 4 and 7 when comparing CINS with healthy participants (Figure 1). This increased IFN-γ expression was only seen on day 4 in SEPSIS patients.

### Relationship between ELISpot measurements and clinical outcomes.

SEPSIS patients had a greater in-hospital, 30-, and 180-day mortality when compared with CINS patients (Table 2). In addition, the incidence of secondary infections, development of CCI, and an adverse discharge disposition were all significantly greater in SEPSIS than CINS patients (all *P* < 0.05).

**Figure 3:**
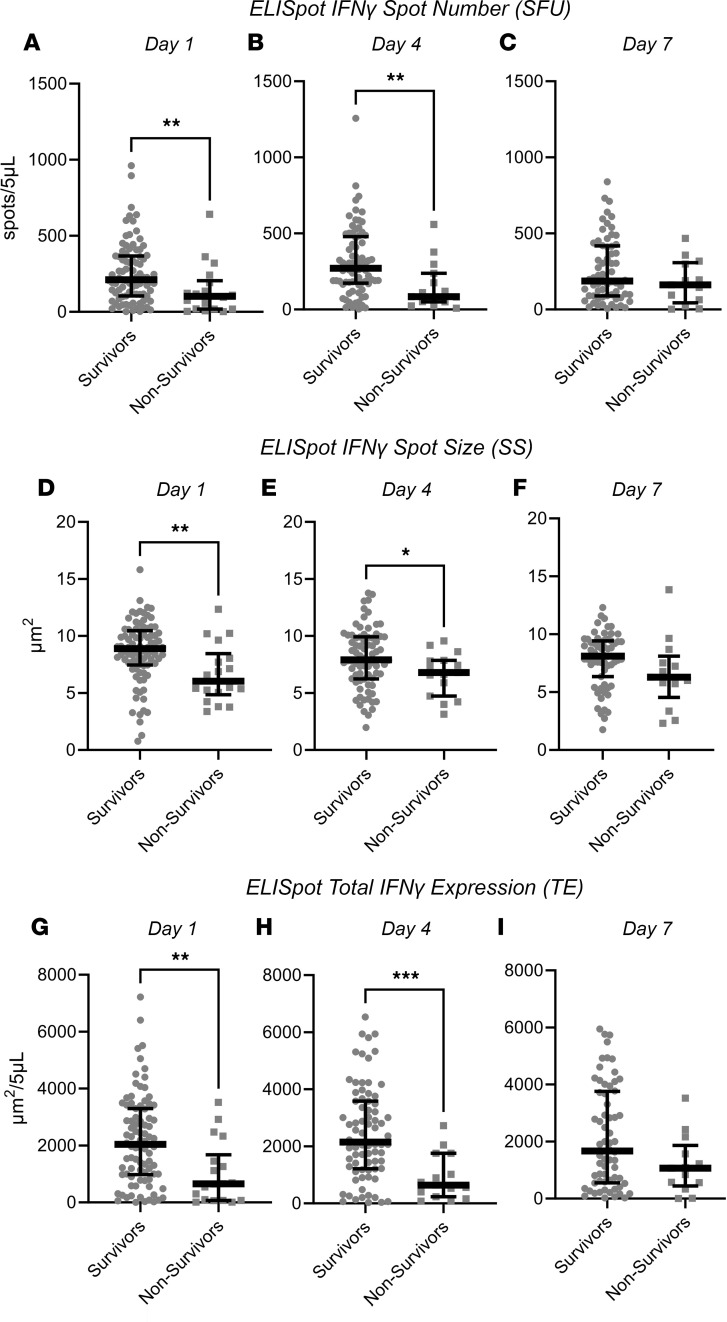
Anti-CD3/anti-CD28–stimulated IFN-γ expression by ELISpot in sepsis patients measured 1, 4, and 7 days after ICU admission who survived or did not survive 180 days. (**A**–**C**) Spot number. (**D**–**F**) Spot size. (**G**–**I**) Total IFN-γ expression. Values represent medians and individual responses. The number of participants declined over time as patients were either discharged from the ICU or died. *P < 0.05, **P < 0.01, ***P < 0.001, as determined by Kruskal-Wallis ANOVA and post hoc analyses using Dunn’s test. Values are 2 sided and represent raw *P* values. SFU, spot-forming units; SS, spot size; TE, total IFN-γ expression.

SEPSIS patients who died within 180 days of ICU admission did not differ from surviving patients based on their admission or day 1 sequential organ failure assessment (SOFA) scores or total leukocyte numbers, even though nonsurviving SEPSIS patients were significantly older and had higher Charlson comorbiditiy scores (both *P* < 0.05; Supplemental Table 2). Interestingly, there were marked differences in the IFN-γ production from stimulated whole blood between sepsis patients who survived or did not survive 180 days. Both the absolute number (SFU) and percentage of IFN-γ–producing cells, and the SS were significantly lower, and therefore, TE was reduced in nonsurviving versus surviving SEPSIS patients (all *P* < 0.05; Figure 3). This reduction in expression was, in general, sustained through day 4 in nonsurvivors (Figure 3). After 7 days, there were too few ICU-remaining patients to continue the comparison (data not shown). Surprisingly, IFN-γ production from unstimulated whole blood did not differ between surviving and nonsurviving patients at any time point (data not shown). There also did not appear to be any significant changes in ELISpot responses (both stimulated and unstimulated) over time in either surviving or nonsurviving individual SEPSIS patients (data not shown).

### Univariate and multivariate prediction models for long-term survival and secondary outcomes.

**Figure 4:**
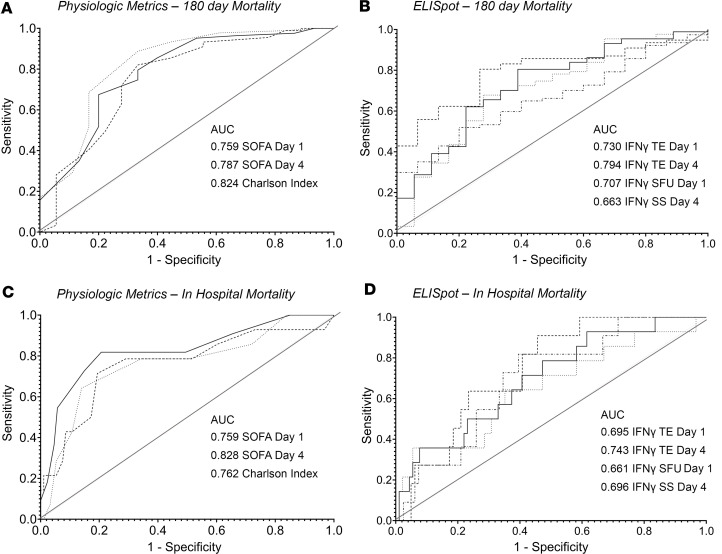
Area under the receiver operator curves (AUROC) for physiologic (SOFA, Charlson comorbidity scores) and stimulated IFN-γ ELISpot responses in differentiating in-hospital and 180-day mortality. (**A**) SOFA and Charlson comorbidity index. (**B**) Selected ELISpot parameters discriminating 180-day mortality. (**C** and **D**) Same as for **A** and **B** but discriminating in-hospital mortality. TE, IFN-γ ELISpot total expression; SFU, IFN-γ ELISpot spot-forming units; SS, IFN-γ ELISpot spot size.

Because of the differences in IFN-γ expression between surviving and nonsurviving SEPSIS patients, ELISpot area under the receiver operating characteristics curve (AUROC) values were evaluated for their discriminatory prediction of long-term survival (180 days), as well as secondary outcomes, and compared to clinical indices (SOFA, Charlson comorbidity scores), total white blood cell and ALCs, and plasma protein markers (selected cytokines, procalcitonin, and soluble PD-L1 [sPD-L1]) in the SEPSIS patients. Similar combined SEPSIS and CINS analyses could not be performed due to the low mortality in CINS patients. Results are presented in Table 3 and Figure 4.

Similar to data reported by ourselves and others (22–24), the most consistent discriminator of 180-day mortality was the Charlson comorbidity score (AUROC 0.824, 95% CI 0.700–0.948), which also discriminated in-hospital mortality (AUROC 0.762, 95% CI 0.611–0.912) and development of CCI (AUROC 0.713, 95% CI 0.594–0.832), but not the incidence of secondary infection (AUROC 0.549, 95% CI 0.426–0.673). Importantly, stimulated total IFN-γ expression on day 1 and day 4 did not significantly differ from the Charlson comorbidity score, with AUROCs of 0.730 (95% CI 0.601–0.859) and 0.794 (95% CI 0.691–0.897), respectively. Total IFN-γ expression on day 4 also discriminated in-hospital mortality, with an AUROC of 0.743 (0.615–0.830), but it was not a strong discriminator of either development of CCI or incidence of secondary infections.

For total stimulated IFN-γ expression, the components contributing to its discriminative power for 180-day mortality were both the number of IFN-γ–producing cells (SFU) (day 1: AUROC 0.707, 95% CI 0.568–0.846) and the amount of IFN-γ produced by individual cells (SS) (day 1: AUROC 0.707, 95% CI 0.568–0.846).

The discriminatory power of the ELISpot TE for predicting both in-hospital and 180-day mortality was much greater than seen for either ALC, IL-6, or procalcitonin on either day 1 or day 4, or for changes in these parameters between days 1 and 7 (Table 3). In addition, the last ELISpot measurement obtained from the patient prior to discharge or death (usually day 7 or later) was also found to be not as discriminatory as the earlier day 1 and 4 measurements (data not shown).

Setting the threshold for day 1 and day 4 stimulated total IFN-γ expression at approximately 80% sensitivity to discriminate 180-day survival, it was possible to assess the immunosuppressive endotype of those SEPSIS patients with reduced ELISpot TE (Table 4). These individuals were older and had ALCs significantly lower at both days 1 and 4 than SEPSIS patients above the ELISpot threshold. In addition, plasma sPD-L1 concentrations were significantly higher on day 4. Patients below the ELISpot threshold also had development of CCI at a markedly higher frequency than those individuals above the threshold (see odds ratios in Table 4).

Finally, to examine whether ELISpot could improve the discriminatory power of standard clinical indices (SOFA and Charlson comorbidity scores), single and multivariate logistic regression analyses were performed and the AUROCs of the models were calculated by a 4-fold cross-validation procedure (Table 5). Model I was built on baseline Charlson comorbidity data and day 1 SOFA scores, yielding AUROCs for 180-day mortality (AUROC 0.911, 95% CI 0.858–0.965); the odds ratios show that baseline Charlson comorbidity data and day 1 SOFA scores are significant predictors for 180-day mortality (Table 5). Model II was built on stimulated total IFN-γ expression from day 4, yielding AUROCs for 180-day mortality (AUROC 0.794, 95% CI 0.732–0.855); the odds ratios show that stimulated total IFN-γ expression is a significant predictor for 180-day mortality (Table 5). Although ELISpot data show significant odds ratios in Model II, the addition of stimulated total IFN-γ expression to the model built on standard score indices (Model III) did not significantly increase either the AUROC or the odds ratio for 180-day (AUROC 0.915, 95% CI 0.851–0.979) or in-hospital mortality (AUROC 0.883, 95% CI 0.801–0.964), or development of CCI (AUROC 0.861, 95% CI 0.830–0.892) (Table 5).

## Discussion

### Key findings.

This prospective, multicenter observational study has demonstrated that the adaptive immune response to critical illness, as defined by ex vivo whole blood production of IFN-γ in response to T cell receptor stimulation, varied in response to critical illness (Figures 1 and 2) and could also discriminate long-term outcomes (Figure 4 and Table 3). Spontaneous IFN-γ production by diluted whole blood was significantly increased in critically ill patients, irrespective of whether the critically ill patients were septic. In addition, when whole blood was stimulated ex vivo with a T cell receptor agonist, IFN-γ production increased dramatically in both healthy and critically ill cohorts (both SEPSIS and CINS). However, stimulated ELISpot IFN-γ TE early in the admission to the ICU significantly differed between SEPSIS patients who survived 180 days and those who died, and this reduction in total expression in nonsurviving SEPSIS patients was due to both reductions in the total number of IFN-γ–producing blood cells and the amount of IFN-γ produced by individual cells (Figure 3). Importantly, day 1 and day 4 measurements were more discriminatory than later measurements. Using univariate modeling, stimulated ELISpot TE measured in the first week of admission (sampling days 1 and 4) could differentiate 180-day mortality as well as SOFA and Charlson comorbidity scores, and markedly better than blood ALC, procalcitonin, IL-6, and sPD-L1 concentrations (Table 3). SEPSIS patients who had low stimulated ELISpot total IFN-γ expression on days 1 and 4 had an immunosuppressed endotype, as reflected in being older, and having lower ALCs, higher plasma sPD-L1, and increased incidence of chronic critical illness, in-hospital mortality, and late mortality (Table 4). However, in multivariate models, stimulated ELISpot total IFN-γ expression did not significantly improve the discrimination between 180-day survival in models built with SOFA and Charlson comorbidity scores (Table 5).

### Context.

ELISpot has emerged as a powerful method to assess immunological status in a variety of clinical disorders (25–28), including septic and critically ill patients (20, 21, 29, 30). It offers several theoretical advantages over other current metrics — for example, cell phenotypes such as ALC (31–34) and HLA-DR expression on CD14^+^ blood cells (35, 36); plasma protein concentrations such as procalcitonin (37–40), IL-6 (41–43), and sPD-L1 (44, 45); or blood transcriptomics (24, 46, 47) — used to predict both the severity of the host response and the immunosuppressed endotype. ELISpot, unlike these static measures, assesses one component of the functional status of the host protective immune response. In the present study, ELISpot revealed the capacity of T cells in the blood to produce IFN-γ with and without stimulation through the T cell receptor. In addition, unlike other assays (such as ELISA or the ELLA automated multiplex ELISA platform), ELISpot can distinguish between the number of cells producing a key cytokine and the amount of cytokine produced by an individual blood cell.

This study is not the first to demonstrate reduced IFN-γ ELISpot expression in septic patients, especially in those with adverse clinical outcomes (20, 21, 48, 49). However, in contrast to these previous studies, we used diluted whole blood in our ELISpot assay instead of isolated PBMCs and observed increased IFN-γ production in both unstimulated and stimulated whole blood from critically ill patients. There are 2 key advantages of using diluted whole blood in the ELISpot. First, the use of whole blood permits the assay to take place with the entire blood composition (i.e., all leukocytes, erythrocytes, platelets, and plasma proteins and metabolites) maintained. Responses to critical illness and ex vivo stimulation may be either direct or be mediated via cell-cell communication and/or plasma mediators. Traditional processing of blood by density gradient centrifugation separates PBMCs from neutrophils, platelets, and plasma. Second, it is a simpler and more rapid assay to set up because there is no required cell isolation step.

On days 1–7, both the CINS and SEPSIS patients had reduced ALC, as compared with healthy controls (Supplemental Figure 1). Surprisingly, despite this lymphopenia, the number of lymphocytes spontaneously producing IFN-γ was higher in both CINS and SEPSIS patients versus healthy controls at all 3 time points (Figure 1). Day 1 stimulated IFN-γ SFU, SS, and TE did not differ between cohorts. However, on day 4, both the number of cells producing IFN-γ as well as TE of IFN-γ was higher in both the CINS and SEPSIS cohorts, as compared with healthy controls. On day 7, the number of cells producing IFN-γ remained higher in the CINS and SEPSIS cohorts. Of note, the amount of IFN-γ produced on a per-cell basis (reflected by SS) was lowest in the SEPSIS cohort, with TE similar to healthy controls.

Given the considerable amount of data showing sepsis can evolve into an immunosuppressed state, it was surprising to see both the spontaneous and stimulated IFN-γ production increase in septic patients (early after admission) versus healthy controls. There are several likely explanations for this apparent paradox. The first potential explanation relates to timing; specifically, the data presented herein came from blood samples collected within the first 7 days after ICU admission. It is difficult to determine exactly when the sepsis-induced hyperinflammation transitions to a state of immunoparalysis, but it is tempting to speculate that our assessment of immune fitness was still within the window of hyperinflammation and exacerbated immune cell activity.

A second explanation has been termed “bystander activation” (50, 51). The inflammatory response that develops during infection has a capacity to trigger antigen-experienced effector and/or memory CD8^+^ T cells present in a T cell receptor–independent and cytokine-dependent manner. A number of cytokines, including IL-12, IL-15, TNF-α, and IL-18 induce CD8^+^ T cell activation and resultant IFN-γ production (52). Thus, the sepsis cytokine milieu likely primes preexisting, effector and memory CD8^+^ T cells to produce IFN-γ in a cognate antigen–independent fashion (Figure 1). In addition, these cytokine-primed effector/memory CD8^+^ T cells will also respond with IFN-γ production to a myriad of cytokines produced ex vivo during anti-CD3/anti-CD28 stimulation.

A third explanation may lie in the differences in the lymphocyte subsets present in the peripheral blood of healthy participants versus CINS and SEPSIS patients at the time of blood collection. De novo clonal expansion of pathogen-specific effector CD8^+^ T cells in response to sepsis-inducing pathogens and resultant inflammation leads to the potential for a preponderance of actively responding effector cells — especially early in the septic timeline. In contrast, healthy control volunteers are more likely to have resting naive and memory T cells and a minimal (if any) increase in inflammatory cytokines. Consequently, the number of T cells capable of rapidly responding to polyclonal and/or bystander cytokine stimulations and produce IFN-γ in the ELISpot assay is increased in SEPSIS patients compared with healthy participants.

### Current work.

The current studies add to the body of information suggesting ELISpot examination of whole blood production of IFN-γ can both discriminate long-term mortality and identify those patients who may benefit from therapeutic interventions targeting adaptive immunity. With that said, 2 questions remain unanswered. The first is the cellular identity of IFN-γ production determined by the ELISpot assay. Although IFN-γ can be produced by a number of blood leukocyte subsets, unpublished data from consortium members suggest T cells, especially memory phenotype T cells, are the principal cells within the blood compartment producing IFN-γ in response to CD3/CD28 ligation. Secondly, the studies do not identify how IFN-γ production is suppressed in nonsurviving SEPSIS patients in response to anti-CD3/anti-CD28 mAb stimulation. Again, data from consortium members and others suggest that blood myeloid-derived suppressor cell (MDSC) numbers are increased in septic patients, with adverse outcomes (20, 53–55). Unpublished findings show the coculture of autologous blood MDSCs from septic patients with T cells activated with anti-CD3/anti-CD28 mAbs suppress not only IFN-γ but also Th1, Th2, and Th17 cytokine production. Such findings reported here suggest the reduced IFN-γ production measured by the ELISpot assay can be due to a reduced number of IFN-γ–producing memory T cells, and/or IFN-γ expression by memory T cells may be actively suppressed, at least in part, by circulating MDSCs.

### Limitations.

This study has several limitations. Despite multicenter enrollment, sample sizes were still relatively small for discriminative modeling. Over the past 2 decades, improved in-hospital management has reduced the number of adverse events and in-hospital mortality to sepsis and critical illness (23, 56). Discriminatory analyses could only be conducted in the SEPSIS cohort, as CINS patients had very low in-hospital (1%) and 180-day mortality (4%) (Table 2). Second, every effort was made to match healthy control participants to the SEPSIS and CINS cohorts, but the healthy donors used in this study were, as a group, significantly younger and more frequently female (Table 1). Median ages in the healthy control cohort were greater than 45 years, a break point often determined to be associated with increased adverse outcomes in critically ill patients (23). Despite the multicentric nature of the study, the cohorts still were also predominantly White. Finally, the SEPSIS and CINS patients were recruited from surgical and trauma ICUs, and therefore represent preponderantly hospital-acquired sepsis. As such, these findings will require confirmation in other sepsis cohorts.

### Future directions.

While the findings presented herein suggest assessing IFN-γ production by ELISpot can be useful in identifying septic patients at risk of long-term mortality and the immunosuppressed endotype, its discriminative ability is similar to that of SOFA and Charlson comorbidity indices and does not add significantly to their discriminative power. With that said, SOFA and Charlson comorbidity indices are rarely used for clinical decision making because they provide no therapeutic directions or insights into the immunological disturbances associated with sepsis and adverse outcomes. Application of ELISpot to the clinical armamentarium has the potential to provide important information regarding which septic patients would benefit from targeted therapy (precision medicine). For example, septic patients who have profound suppression of stimulated IFN-γ production may be harmed by therapy with corticosteroids, but might be good candidates for immune-adjuvant therapies to boost their ability to combat invading pathogens. ELISpot is an FDA-cleared approach for assessing functional immune status to prior tuberculosis infection and the ELISpot reader used in these studies (CTL S6 Entry) is FDA 510(k) cleared. However, to make these results more actionable, ELISpot results will need to be obtained within hours, instead of days. Currently, ELISpot results take at least 24 hours to return, although preliminary data from our consortium suggest the assay can be modified to produce results in less than 12 hours (TSG and CCC, unpublished observations).

In addition, ELISpot can be readily used to assess other components of the blood innate and adaptive immune response simply by varying the stimulant and the readout metric. For example, innate immune responses have been readily assessed using endotoxin or other TLR ligands as a whole-blood stimulant and TNF-α as the readout (20, 21). Furthermore, underlying mechanisms of adaptive or innate immune responses can be explored using alternative stimulants (28, 29, 57), simultaneous adjuvants or inhibitors (58), or different readout metrics (21, 57).

### Conclusions.

ELISpot can assess functional immune status in critically ill patients, predict adverse long-term outcomes, and identify subsets of patients who may benefit from immunostimulant therapy**.**

## Methods

**Figure 5:**
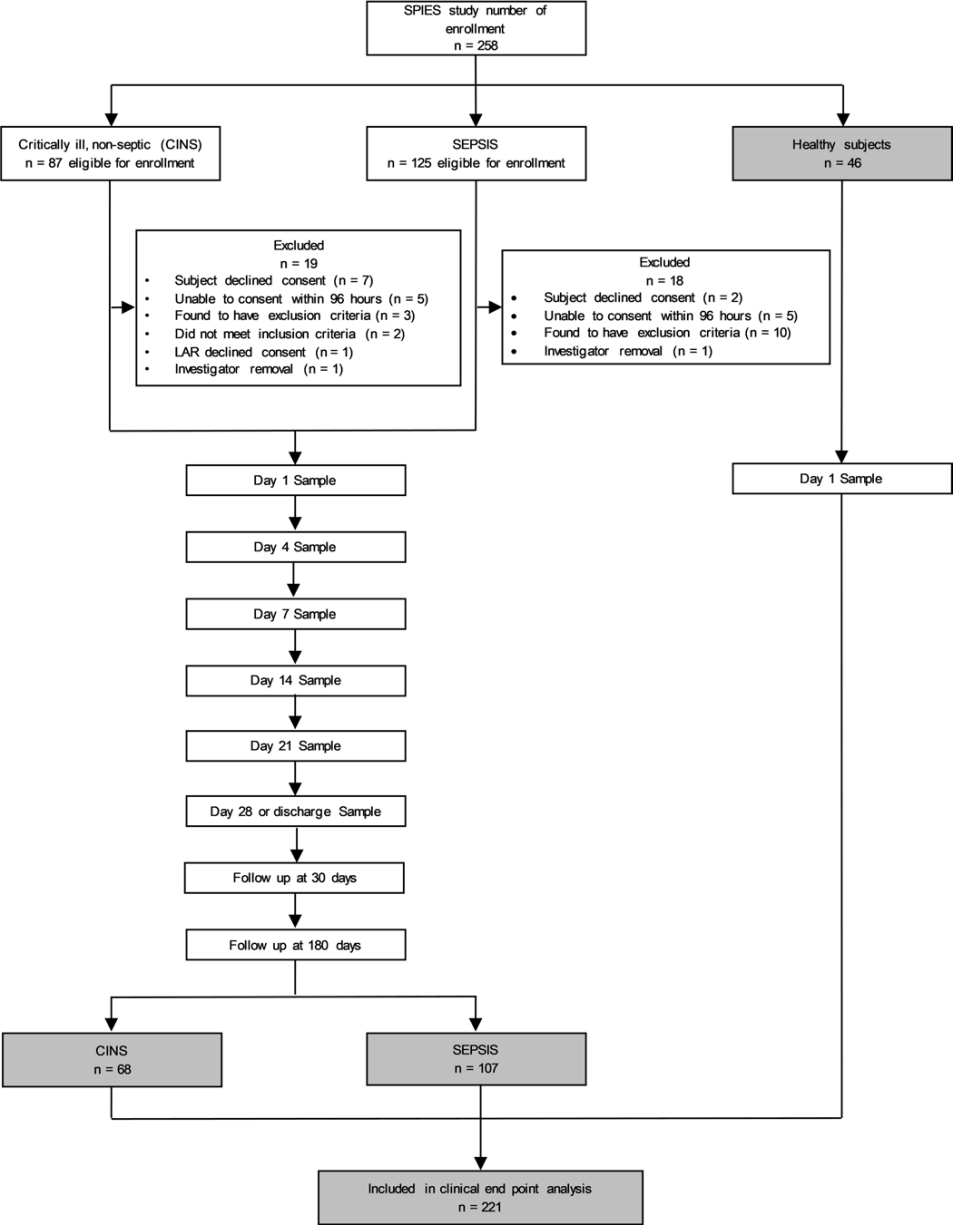
Flow diagram for study enrollment. SPIES, Stratifying Patient Immune Endotypes in Sepsis study.

This multicenter, prospective diagnostic and prognostic study, conducted between February 23, 2021, and July 22, 2022, enrolled 2 cohorts of critically ill patients at the time of ICU admission. The first cohort included patients with a suspected diagnosis of sepsis admitted to the surgical/trauma ICU (SEPSIS). The second cohort included critically ill patients admitted to the ICU without currently suspected sepsis (CINS), but considered at high risk for subsequent infection (e.g., postoperative, severe trauma). Patient enrollment is shown in Figure 5, consistent with Enhancing the Quality and Transparency of Health Research Standards for Reporting of Diagnostic Accuracy (STARD) reporting guidelines (59). All patients were managed under institutional clinical management protocols.

Blood was obtained using heparinized blood collection tubes (Becton Dickinson) within the first 3 days of ICU admission (labeled as day 1), and on subsequent days 3 through 5 (labeled as day 4), and weekly thereafter (±2 days). Self- or proxy-reported race and ethnicity category data were collected as per NIH reporting guidelines and requirements.

Inclusion criteria consisted primarily of ICU admission with sepsis from either severe trauma, nontrauma, postoperative ICU admission, ICU transfer from the emergency department, and inpatient transfer from ward to ICU (see Supplemental Table 1 for admission reasons). A small proportion of patients were admitted directly from the emergency department with suspicion of community-acquired sepsis.

Sepsis was defined according to Sepsis-3 criteria (4), and all participants were clinically adjudicated at the individual participating sites. Patients admitted to the ICU for CINS were also adjudicated to rule out sepsis. A detailed summary of inclusion and exclusion criteria is provided in the Supplemental Methods. Individual criteria for inclusion as CINS and sources of infection in the SEPSIS cohort are summarized in Supplemental Table 1.

Healthy control participants were recruited at each of the clinical sites. Efforts were made to match the healthy control participants’ age, sex, and race/ethnicity to those of the SEPSIS cohort. Individuals with autoimmune diseases being treated with biologic immune modulators were excluded, as were individuals who had received antineoplastic therapies or diagnosed with cancer within the previous 6 months. Vulnerable populations were also excluded.

### Primary outcomes and clinical adjudication.

The primary clinical outcome for ELISpot was 180-day mortality, determined via clinical records and telephone follow-up with the patient, their proxy, or their designated contact, and cross-checked through the US Social Security Death Index. We analyzed temporal trends of ELISpot in both SEPSIS and CINS patients but compared estimated performance of predictive models primarily in the SEPSIS patients, as 180-day mortality in the CINS cohort was less than 4%. Final SEPSIS or CINS adjudication was performed by individual physician-investigators at each clinical site at completion of each patient’s hospital course according to Sepsis-3 criteria. Over the course of the study, 18 patients initially assigned to SEPSIS were adjudicated as CINS, and 6 CINS patients were adjudicated as SEPSIS.

Secondary clinical outcome variables included all-cause (in hospital, 30-day) mortality, development of CCI, secondary infections, and poor discharge disposition. Inpatient clinical trajectory was defined as “early death,” “rapid recovery,” or “CCI.” CCI was defined as an ICU length of stay of 14 or more days with evidence of persistent organ dysfunction (SOFA score ≥ 2) (60). Hospitalized patients who died after an ICU length of stay greater than 14 days from the index hospitalization were also classified as CCI. Poor disposition was defined as discharge to a skilled nursing facility, long-term acute care facility, or hospice. Secondary infections were defined as per the US Centers for Disease Control and Prevention criteria.

### ELISpot.

ELISpot assays were conducted using the human IFN-γ Immunospot kit (CTL Inc.) with several important modifications, including the use of diluted whole blood as previously described (21). Specifically, 100 mL of heparinized whole blood was diluted 1:10 with kit buffer and 50 mL of the diluted sample was added to each well. Samples were incubated in wells containing either buffer alone or a soluble anti-CD3/anti-CD28 (125 ng/mL and 1.25 μg/mL, respectively) mAb agonist (BioLegend). Samples were assayed in duplicate. Optimal concentrations of agonist were determined in preliminary studies (see Supplemental Methods).

Samples were quantified using a CTL S6 Entry or S6 FluoroCore ELISpot reader at each clinical site. To assure comparable results, the instruments were harmonized by CTL Inc. prior to the study using an external standard across all 5 clinical sites. Results are presented as the number of SFU, SS (μm^2^), and TE (μm^2^), a product of the number of spots and mean spot size using the Immunospot SC software suite (version 7.0.30.4). SFU represents individual blood cells expressing IFN-γ and SS is an indication of the amount of IFN-γ produced per cell. In subsequent analyses, the number of IFN-γ–producing cells was adjusted for each individual patient’s ALC to yield the percentage of total lymphocytes expressing IFN-γ.

### Additional laboratory analyses.

Whole-blood total leukocyte counts and ALCs were determined on EDTA-anticoagulated whole blood at the individual clinical sites either using their hospital’s Clinical and Diagnostics Laboratory or a research Beckman-Coulter Dx500 or Dx900 hemocytometer. Cytokine and additional plasma protein analyses were conducted at the University of Florida Sepsis and Critical Illness Research Center (SCIRC) where they were determined in batch using the Luminex MagPix platform with commercial reagents.

### Data collection and analysis.

Clinical data collection was conducted at each site and entered into a web-based electronic case report form created on the REDCap platform managed by the University of Florida Clinical and Translational Science Institute (CTSI). Access to the case report form was password protected and limited to only approved research staff, and all interactions with the database were recorded. Peer-to-peer communication allowed approved individuals at all 5 sites access to their own data and deidentified data from the other 4 clinical sites. Research data, including ELISpot, total leukocyte counts and ALCs, and plasma protein and cytokine data were uploaded into the case report forms from the University of Florida SCIRC. Data managers at the SCIRC were responsible for creating final locked data sets for subsequent analysis.

### Statistics.

Descriptive data are presented as frequencies and percentages, medians and interquartile ranges, and means and standard deviations, where indicated. Fisher’s exact test and Mann-Whitney or Kruskal-Wallis ANOVA tests were used for categorical and continuous variables, respectively. AUROC values with 95% CIs (computed with 2000 stratified bootstrap replicates) were used to assess discrimination. Univariable and multivariable logistic regression were performed to assess whether the combination of metrics improved overall performance. Post hoc tests were performed for continuous outcomes using Dunn’s test. For post hoc analyses of categorical outcomes, separate 2 × 2 Fisher exact tests were performed. All significance tests were 2-sided, with a raw *P* value of 0.05 or less considered statistically significant. Analyses were performed using the R Project statistical package, version 4.1.0 (R Project for Statistical Computing; https://www.r-project.org/).

### Study approval.

Centralized ethics approval was obtained from the University of Florida Institutional Review Board (IRB 202000924), which served as the sponsoring institution for this multicenter clinical study. Written informed consent was obtained from each patient or their proxy decision maker at individual clinical sites.

### Data availability.

Deidentified clinical data and excess plasma samples are stored at the Biorepository of the Clinical and Translational Science Institute (https://www.ctsi.ufl.edu/research/laboratory-services/ctsi-biorepository-2/) where it is available to the scientific community under guidelines promulgated by the National Institute of General Medical Sciences (NIGMS). Data associated with the main manuscript and supplement material — including values for all data points shown in graphs and values behind any reported means — are available in the Supporting Data Values Excel (XLS) in the supplemental material.

## Author contributions

ELB performed experiments, generated figures, and reviewed and edited the manuscript. PAE provided resources and supervision, acquired data and funding, and reviewed and edited the manuscript. ML, VEP, EES, MH, MR, TAK, MLSD, LZS, and JX performed experiments and generated figures. LEB and TJL provided supervision, performed experiments, and reviewed and edited the manuscript. LLM conceptualized the study, acquired data and funding, provided resources, supervision, and project administration, and reviewed and edited the manuscript. RFU supervised the project, performed experiments, and generated figures. JPL supervised the project and conducted experiments. RJD, AEM, DDT, AHW, AMD, S Meszaros, and S Miles performed experiments. PWM and RWG conceptualized the project and provided project administration. VPB, TSG, RSH, and CCC conceptualized the study, acquired funding and data, provided resources and supervision, and reviewed and edited the manuscript. CYK and CBB performed experiments and acquired data. LOH and CRN provided project administration. IJT conceptualized and supervised the study, and reviewed and edited the manuscript. SCB conceptualized the study, acquired funding, and reviewed and edited the manuscript. MDG acquired data, performed experiments, generated figures, and provided supervision. MBM conceptualized and supervised the study, conducted experiments, and reviewed and edited the manuscript. KER conceptualized the study, performed experiments, acquired data, and reviewed and edited the manuscript. ILR acquired data, edited the manuscript, and analyzed data.

## Supplementary Material

Supplemental data

ICMJE disclosure forms

Supporting data values

## Figures and Tables

**Table 1:**
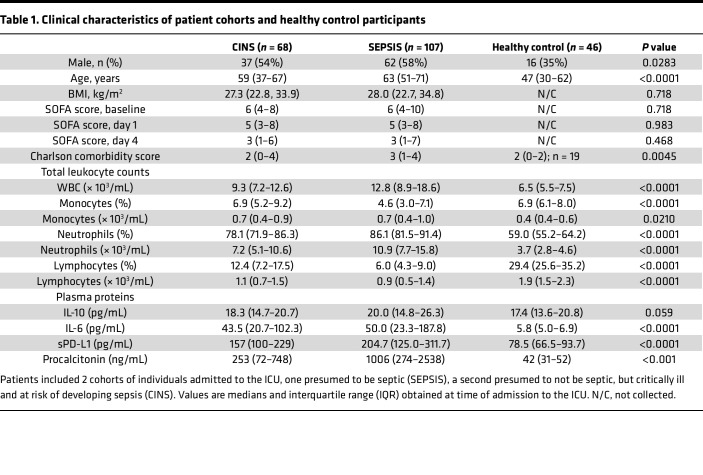
Clinical characteristics of patient cohorts and healthy control participants

**Table 2:**
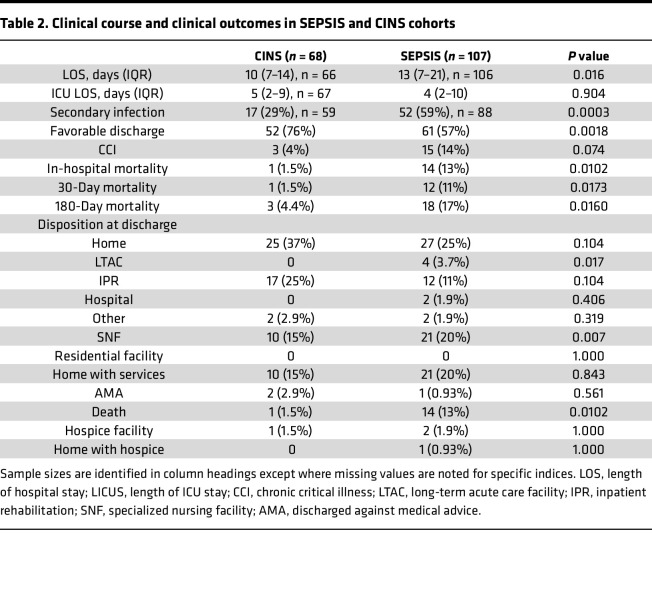
Clinical course and clinical outcomes in SEPSIS and CINS cohorts

**Table 3:**
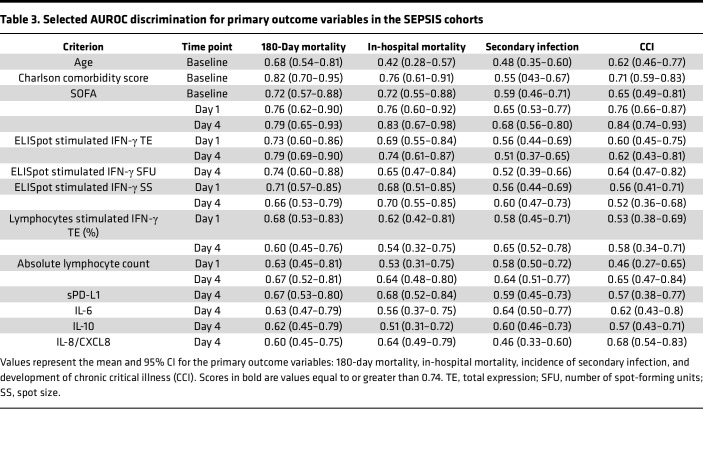
Selected AUROC discrimination for primary outcome variables in the SEPSIS cohorts

**Table 4:**
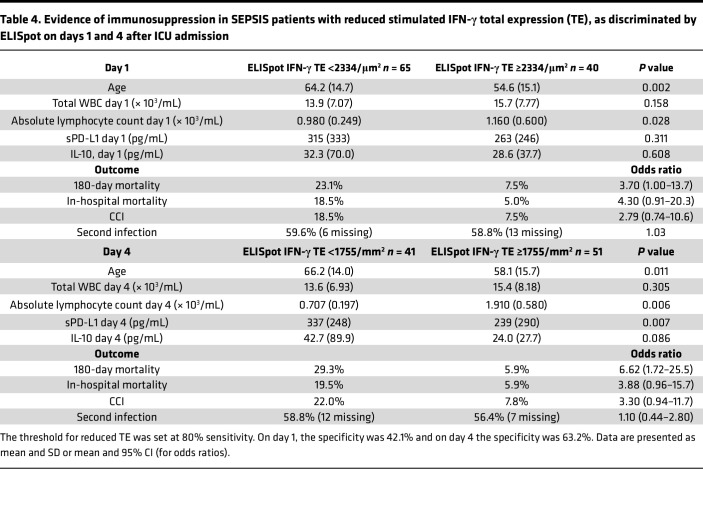
Evidence of immunosuppression in SEPSIS patients with reduced stimulated IFN-γ total expression (TE), as discriminated by ELISpot on days 1 and 4 after ICU admission

**Table 5:**
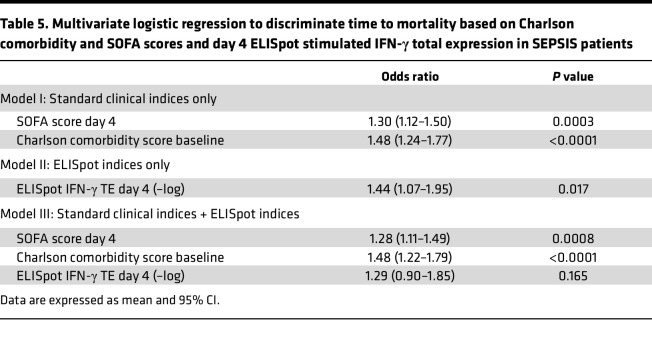
Multivariate logistic regression to discriminate time to mortality based on Charlson comorbidity and SOFA scores and day 4 ELISpot stimulated IFN-γ total expression in SEPSIS patients

## Version 1 12/15/2023

In-Press Preview

## Version 2 01/23/2024

Electronic publication
