# Supplementary material for: Adverse outcomes and an immunosuppressed endotype in septic patients with reduced IFN-γ ELISpot
Source: JCI Insight. 2024 Jan 23;9(2):e175785. doi: 10.1172/jci.insight.175785 (PMC10906237; doi:10.1172/jci.insight.175785)

Supplemental Methods and Results

Detailed specific criteria for consenting and enrolling SEPSIS and CINS patients, and healthy control subjects are described below, as is sampling and processing procedures. Actual IRB documentation and the Consortium Laboratory Manual are readily available by simply requesting them from our data and sample broker, the UF Clinical and Translational Research Institute Biorepository (https://www.ctsi.ufl.edu/research/laboratory-services/ctsi-biorepository-2/scirc-specimens-archive/).

Recruitment methods

Screening for sepsis will be carried out using each hospital's own version of their sepsis alert system, which quantifies derangements in vital signs, white blood cell count, and mental status. After a putative diagnosis of sepsis, the patient is transferred to the ICU and sepsis treatment bundles are initiated. If a patient is believed to have an infection and they are located in, or transferred to the ICU, they are entered into each institution's sepsis management protocol as standard of care which implements a variety of standard operating procedures (SOPs) of clinical ICU care. After the patient enters the clinical management protocol, the research coordinator is notified of a potential research subject. This coordinator will then assess the following inclusion/exclusion criteria and consent the appropriate candidates.

All critically ill patients with or without sepsis will be managed via each institution's evidence-based management protocols that emphasize early antibiotic administration, fluid resuscitation and hemodynamic monitoring and support, consistent with current Surviving Sepsis Campaign guidelines.

Consenting

Consent will be sought by clinical research staff, all of whom are familiar with institutional logistics and infrastructure, sample acquisition and preparation, and are experienced in the nuances of enrollment and informed consent for this challenging patient population. Many critically ill patients in the ICUs may have altered mental status or pharmacologic sedation but would be regarded as prospective research subjects. We will seek an IRB-approved 96-hour delayed consent for blood sample acquisition and completion of the /T1 visit per protocol (within the first 72 hours). This includes: study criteria evaluation, sample collection and transportation to clinical and research laboratories, collection of demographic information, and collection of medical laboratory results to compliment research experiments. Additionally, we will seek approval for telephone consent in the event that no LAR is physically present. For the ICU patient population, many of whom are pharmacologically sedated and mechanically ventilated, initial consent is commonly requested from LAR/next of kin. The setting for conveying consent information to LAR/next of kin is often the ICU family waiting area or at bedside. Consideration is given to the emotional status of the LAR/next of kin and ability to understand the basic intent, methods, and voluntary nature of participation. If we need to call a LAR, ideally, we will email them a copy of the consent and review, record approval with a witness on the line. If we can’t email them, then we will review the entire consent over the phone, record approval with a witness on the line.

The research staff will monitor the patient’s progress and once they regain capacity, the patient will be re-consented. If patient does not regain capacity to consent by discharge, he/she will be consented at their next follow-up visit if capacity is regained.

For the healthy control subjects, we will send an email out to the Department of Surgery email distribution list to all department staff for recruiting healthy control participants. When the participant comes in for the blood draw we will review the consent and have them sign.

Specific Inclusion/Exclusion Criteria

SEPSIS- Presumed Sepsis Patients

Inclusion Criteria

A. Directly admitted ICU patients with sepsis (From ED or OR)
   ICU patient developing sepsis during hospitalization.
   Transferred to ICU from inpatient unit for development of sepsis.
   Documentation in notes or diagnoses of "Sepsis", "Septic shock", "Severe Sepsis"

or

B. Suspicion of infectious cause of illness on admission
   Documentation of suspected infection
   Infectious testing performed (blood, urine, other cultures, viral or fungal testing)
   Diagnostic testing: chest X-ray. CT scan of abdomen
   Initiation of antimicrobial therapy (antibiotics, antivirals, antifungals)
   Source control operation/procedure performed

and

Organ dysfunction as defined as an acute change in total SOFA score of 2 points consequent to the infection.
The baseline SOFA score can be assumed to be zero in patients not known to have preexisting organ dysfunction.

Exclusion Criteria

Patients deemed to be futile care or have advanced care directives or goals of care limiting resuscitative efforts.
Severe traumatic brain injury (evidence of neurologic injury on CT scan and a GCS <8 after resuscitation).
Refractory shock (i.e., patients who are expected to die within 24hours).
Uncontrollable source of sepsis (e.g. irreversible disease state such as unresectable dead bowel).
Outside facility transfer where suspected sepsis onset is >72 hours prior to ICU admission.
Active chronic hepatitis or other chronic infectious diseases.
Known HIV infection with CD4 count <200 cells/mm^3.
Organ transplant recipient on immunosuppressive agents.
Known pregnancy.
Prisoners.

Institutionalized patients.
Active cancer.
Any recent (past 6 months) chemotherapy or immunomodulatory therapies (including biologics, monoclonal antibodies).
Patient having received steroids in past 4 weeks.

CINS – Critically-ill non-sepsis patients

Inclusion Criteria

A. Patients admitted to the SICU for non-infectious reasons
Trauma patients
Post-operative patients (not infectious source control procedures)
Inpatients transferred to the SICU for non-infectious reasons (e.g., bleeding, volume overload, acute cardiac issue, ect.)

Exclusion criteria

Patients deemed to be futile care or have advanced care directives or goals of care limiting resuscitative efforts.
Severe traumatic brain injury (evidence of neurologic injury on CT scan and a GCS <8 after resuscitation).
Refractory shock (i.e., patients who are expected to die within 24hours).
Uncontrollable source of sepsis (e.g. irreversible disease state such as unresectable dead bowel).
Outside facility transfer where suspected sepsis onset is >72 hours prior to ICU admission.
Active chronic Hepatitis or other chronic infectious diseases.
Known HIV infection with CD4 count <200 cells/mm3.
Organ transplant recipient on immunosuppressive agents.
Known pregnancy.
Prisoners.
Institutionalized patients.
Active cancer.
Any recent (past 6 months) chemotherapy or immunomodulatory therapies (including biologics, monoclonal antibodies).
Patient having received steroids in past 4 weeks.

Healthy Control Subjects

Inclusion Criteria

All adults (age >=18)
Ability to obtain Informed Consent prior to blood collection.

Exclusion Criteria

Current, chronic steroid use
Pregnancy
Current or recent (within 7 days) use of antibiotics.

Sample Collection

The overview of research sample collection for SPIES clinical study are presented in the Sample Collection Chart:

SPIES Sample Collection Chart						
	**T1**<br>**Day 1**	**T2**<br>**Day 4**<br>**(+/- 1)**	**T3**<br>**Day 7**<br>**(+/- 1)**	**T4**<br>**Day 14**<br>**(+/- 2)**	**T5**<br>**Day 21**<br>**(+/- 2)**	**T6**<br>**Day 28 or discharge**<br>**(+/- 2)**
**Whole Blood ELISpot (1ml), HLA-DR (1ml), Cytokines (4-8ml) - 6-10ml heparin (green)**	X	X	X	X	X	X
**(Optional) CBC w/diff - 2ml EDTA (purple)**	X	X	X	X	X	X
**Gene Expression - 2.5ml PAXgene**	X	X	X	X	X	X
**Total blood (ml)**	**8.5 - 14.5**	**8.5 - 14.5**	**8.5 - 14.5**	**8.5 - 14.5**	**8.5 - 14.5**	**8.5 - 14.5**

PLASMA PROCESSING

Administrative Note: All personnel involved in the procedures are to have completed the University required Blood-Borne Pathogen training program, and provided suitable personal protection equipment (PPE). These procedures are to be performed whenever possible in a biocontainment hood (BSL1 or BSL2). All solid or liquid waste derived from the blood must be disposed of appropriately in a biohazard container or inactivated using bleach or an acceptable disinfectant. Venous or arterial whole blood is collected into the appropriate blood collection tubes for each time point. The collection of blood should be obtained from an existing arterial or venous line, or venipuncture should be performed by someone experienced in the

technique, and familiar with infectious precautions. The blood should be processed as soon as possible, **but within 3 hours of the draw**. The blood should be **kept cold on ice** during the period from the draw to the initiation of processing.

CBC with Differential

Each site must collect for and obtain a CBC with Differential at every patient sample time point. These must be collected at the same time as the study blood draws or as close to it as possible if done through the hospital's standard of care. This data must be entered into REDCap.

Plasma Collection

1. Invert the green heparin blood tube gently to thoroughly mix the blood. Transfer one ml of blood to a 15 ml conical (polyethylene or polystyrene) tube labeled HLA-DR. And transfer 500 ul of blood to a 1.5 ml microcentrifuge tube labeled ELISpot.

2. After removing the blood needed for the ELISpot and Smart Tube assays in Step 1, centrifuge the remaining blood in the green top heparin blood collection tube at room temperature (22°C) for 10 minutes at 1,800 x g, with the brake on low.

3. Once the tube has finished spinning, carefully transfer the plasma from the green top blood tube (take care not to disturb the cellular constituents) and aliquot 500 ul of plasma equally into 4-6 **green** capped plasma collection tubes. If there is any remaining plasma, collect and distribute evenly among all tubes.

4. Make sure the appropriate *Heparin Plasma* barcode labels are attached to the tubes and immediately store at -80°C.

Supplementary Table 1. Admission Reasons for SEPSIS and CINS Cohorts.

**Sepsis Cohort**	**Abdominal**	**Pulmonary**	**Skin/Soft Tissue**	**Genitourinary**	**Other[1]**	**Total**
**Source of Infection**	35	19	29	12	11	107

**CINS Cohort**	**Surgery not Specified**	**Trauma**	**Pancreatitis**	**Vascular Limb Ischemia**	**Other[2]**	**Total**
**Reason for Admission**	5	33	2	1	27	68

[1] taken from electronic case report form, as other

Osteomyelitis
Ludwig's angina (4)
Acute epiglottis
Frostbite
Bacteremia, shock (multiple)
Septic arthritis (knee)

[2] taken from electronic case report form, as other

Ventral hernia w/out obstruction or gangrene
Facial abscess with necrosis
Non-bleeding duodenal ulcer and significant erosive esophagitis and gastritis
Hypothermia
Pulmonary edema
Acute hypoxic respiratory failure
Seizure
Post-operative intubation
Ischemic bowel disease
Cardiac arrest
Chronic systolic heart failure
Diabetic ketoacidosis without coma associated with type 2 diabetes mellitus
Peritoneal dialysis catheter malfunction
Shock
Cardiac arrest with concern for non-convulsive status epilepticus
Acute renal failure and hypotension
Retroperitoneal bleed
Acute mesenteric ischemia
Undifferentiated shock and refractory hypoglycemia
Seizure
Respiratory Failure

Supplementary Table 2. Demographics and Outcomes Between SEPSIS Patients who Survived or Died Within 180 Days after Sepsis. Values represent the number of sample measurements for each analyte.

	**Survivors, n=89**	**Non-Survivors, n=18**	**p value**
**Male [n (%)]**	53 (60%)	9 (50%)	0.454
**Age, years**	62 (48, 70)	66 (59, 80)	**0.0166**
**BMI, kg/m^2**	28.0 (22.8, 34.9)	25.5 (21.3, 34.6)	0.490
**SOFA Score, Baseline**	6 (4, 8)	10 (5, 11)	**0.0236**
**Charlson Comorbidity Score**	2 (1, 4)	6 (4, 7)	**<0.0001**
**Total Leukocyte Counts**	**Survivors, n=82**	**Non-Survivors, n=17**	
**WBC ($x10^3/\mu l$)**	12.9 (9.6, 19.0), n=85	11.0 (8.1, 17.0), n=18	0.371
**Monocytes (%)**	4.6 (3.1, 7.5), n=84	4.7 (2.6, 6.0)	0.645
**Monocytes ($x10^3/\mu l$)**	0.7 (0.4, 1.0)	0.4 (0.3, 1.0)	0.264
**Neutrophils (%)**	86.1 (81.5, 91.4), n=81	85.0 (80.6, 90.9)	0.729
**Neutrophils ($x10^3/\mu l$)**	11.0 (8.0, 17.0)	8.8 (6.8, 13.4)	0.158
**Lymphocytes (%)**	6.5 (4.3, 9.4), n=84	5.9 (5.3, 8.5)	0.980
**Lymphocytes ($x10^3/\mu l$)**	0.8 (0.5, 1.2), n=84	0.7 (0.5, 1.3)	0.684
**Plasma Proteins**	**Survivors,n=86**	**Non-Survivors, n=18**	
IL-10 (pg/ml)	19.7 (14.8, 26.3)	21.2 (16.3, 29.1)	0.578
IL-6 (pg/ml)	48.9 (20.0, 187.9)	60.3 (25.9, 149.2)	0.730
sPD-L1 (pg/ml)	190 (117, 303)	275 (181, 403)	0.144
Procalcitonin (ng/ml)	993 (254, 3254)	1106 (298, 1810)	0.487
**Clinical Outcomes**	**Survivors, n=89**	**Non-Survivors, n=18**	**p value**

**LOS, days**	13 (8, 20), n=88	14 (7, 20)	0.812
**ICU LOS, days**	4 (2, 9)	6 (2, 15)	0.551
**Secondary infection**	42 (58%), n=73	10 (67%), n=15	0.512
**Favorable discharge**	59 (66%)	2 (11%)	**0.00002**
**CCI**	10 (11%)	5 (28%)	0.0652
**In-Hospital Mortality**	1 (1%)	13 (72%)	**<0.00001**
**30-Day Mortality**	0	12 (70%)	**<0.00001**
**180-Day Mortality**	0	18 (100%)	
**Disposition at discharge**			**0.035**
**Home**	26 (29%)	1 (6%)	
**LTAC**	4 (4%)	0	0.404
**IPR**	11 (12%)	1 (6%)	
**Hospital**	2 (2%)	0	
**Other**	2 (2%)	0	0.319
**SNF**	19 (21%)	2 (11%)	
**Residential facility**	0	0	
**Home with services**	21 (24%)	0	**<0.00001**
**AMA**	1 (1%)	0	
**Death**	1 (1%)	13 (72%)	
**Hospice facility**	2 (2%)	0	
**Home with hospice**	0	1 (6%)	

Supplemental Figure 1. Absolute Lymphocyte Counts in SEPSIS and CINS Cohorts at Different Time Intervals and Healthy Subjects. Whole blood was collected at different time points and total and absolute lymphocyte counts were determined. Both SEPSIS and CINS resulted in a significant decline in total lymphocytes when compared to healthy subjects on days 1, 4 and 7, while absolute lymphocyte counts were lower in SEPSIS than in CINS patients on day 1. Healthy control subjects were sampled only once but values are presented at each time point for comparison. * p<0.05, *** p<0.001, **** p<0.0001 as determined by Kruskal-Wallace ANOVA and post-hoc analyses using the Dunn test. Values are two sided and represent raw p values.

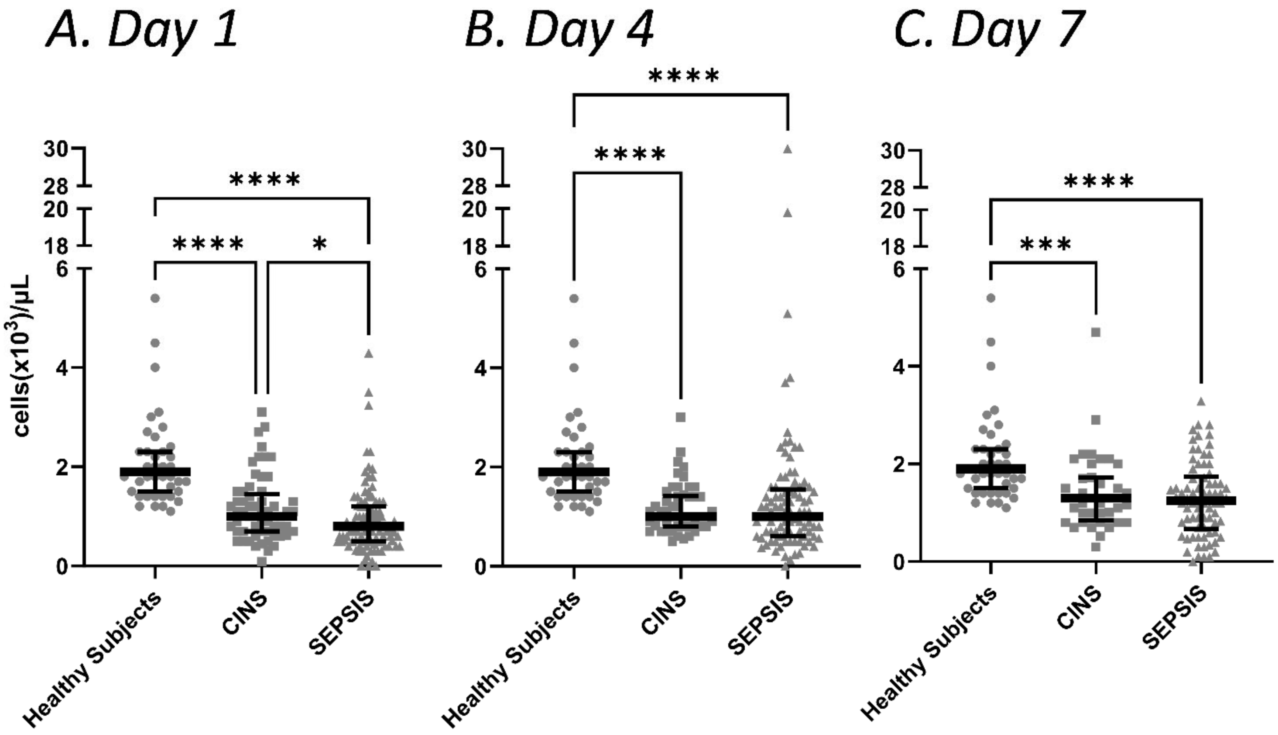

Supplemental Figure 2. Percentage of Lymphocytes Expressing IFNγ in Whole Blood from SEPSIS and CINS cohorts, and Healthy Subjects. The number of spot forming units was compared to the absolute lymphocyte count and the percentage of lymphocytes producing IFNγ was calculated. Regardless of time after enrollment, both SEPSIS and CINS markedly increased the percentage of IFNγ-producing cells. * $p<0.05$, *** $p<0.001$, **** $p<0.0001$, as determined by Kruskal-Wallace ANOVA and post-hoc analyses using the Dunn test. Values are two sided and represent raw p values. SFU, spot forming units. SS, spot size. TE, total IFNγ expression.

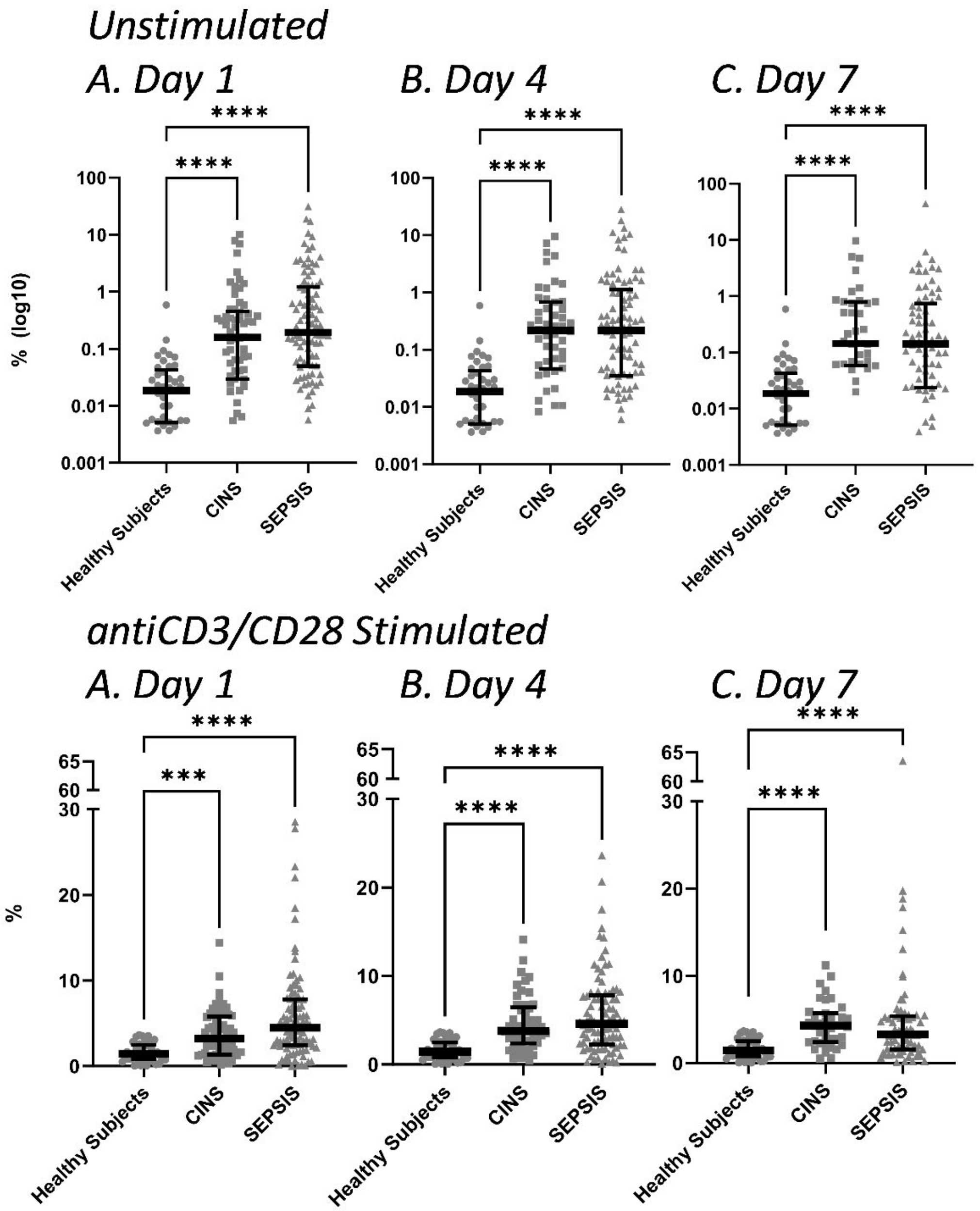

Supplement: Supplemental data [file jciinsight-9-175785-s235.pdf]
